# Associations of Household and Neighborhood Contexts and Hair Cortisol Among Mexican-Origin Adolescents From Low-Income Immigrant Families

**DOI:** 10.1002/dev.22519

**Published:** 2024-09

**Authors:** Ka I Ip, Wen Wen, Lester Sim, Shanting Chen, Su Yeong Kim

**Affiliations:** 1 Institute of Child Development, University of Minnesota, Minneapolis, Minnesota, USA; 2 Department of Human Development and Family Sciences, University of Texas at Austin, Austin, Texas, USA; 3 School of Social Sciences, Singapore Management University, Singapore, Singapore; 4 Department of Psychology, University of Florida, Florida City, Florida, USA

**Keywords:** adolescents, hair cortisol, HPA axis, immigrant, neighborhood socioeconomic status, racial-ethnic concentration, stress

## Abstract

Although neighborhood contexts serve as upstream determinants of health, it remains unclear how these contexts “get under the skin” of Mexican-origin youth, who are disproportionately concentrated in highly disadvantaged yet co-ethnic neighborhoods. The current study examines the associations between household and neighborhood socioeconomic status (SES), neighborhood racial–ethnic and immigrant composition, and hair cortisol concentration (HCC)—a physiological index of chronic stress response—among Mexican-origin adolescents from low-income immigrant families in the United States. A total of 297 (54.20% female; mage = 17.61, SD = 0.93) Mexican-origin adolescents had their hair cortisol collected, and their residential addresses were geocoded and merged with the American Community Survey. Neighborhoods with higher Hispanic-origin and foreign-born residents were associated with higher neighborhood disadvantage, whereas neighborhoods with higher non-Hispanic White and domestic-born residents were associated with higher neighborhood affluence. Mexican-origin adolescents living in neighborhoods with a higher proportion of Hispanic-origin residents showed *lower* levels of HCC, consistent with the role of the ethnic enclave. In contrast, adolescents living in more affluent neighborhoods showed *higher* levels of HCC, possibly reflecting a physiological toll. No association was found between household SES and HCC. Our findings underscore the importance of taking sociocultural contexts and person–environment fit into consideration when understanding how neighborhoods influence adolescents’ stress physiology.

## 1 ∣ Introduction

Residential segregation, a key aspect of social organization in the United States, reflects both classism and structural racism in the housing market (Williams and Collins 2001). Families navigate these constraints and exercise agency in selecting neighborhoods aligned with their preferences, including the presence of co-ethnics. Segregation gives rise to two neighborhood phenomena: Communities of color are disproportionately patterned into neighborhoods with higher concentrations of poverty and socioeconomic disadvantage, and neighborhoods characterized by predominantly non-Hispanic Whites or communities of color. Specifically, influenced by structural racism like residential segregation and limited intergenerational wealth, Mexican immigrant families in the United States often experience high poverty rates (Lopez and Velasco 2011) and are concentrated in lower socioeconomic or higher co-ethnic neighborhoods (Massey 1996; Reardon et al. 2015). Because neighborhoods significantly impact adolescents’ ecological environment, the socioeconomic and racial–ethnic structural characteristics of their residential neighborhoods could have downstream health consequences beyond household factors such as family income (Ip et al. 2022; Leventhal and Dupéré 2019; Sampson, Raudenbush, and Earls 1997; White et al. 2020).

Although prominent theories (e.g., Massey 2004; Roosa et al. 2009; Rumbaut and Portes 2001; Sampson 2002; White et al. 2018) propose understanding how neighborhood contexts may associate with adolescents’ stress physiology, empirical studies examining how neighborhood contexts influence the stress-related hypothalamic–pituitary–adrenal (HPA) axis, especially among racial–ethnic minoritized adolescents (i.e., Mexican-origin youth), are lacking (Harnett 2020). The current study aims to address this knowledge gap by exploring associations between neighborhood socioeconomic status (SES) (including socioeconomic disadvantage and affluence), neighborhood racial–ethnic and immigrant structuring, and hair cortisol concentration (HCC). HCC serves as a physiological index of chronic stress response in the HPA axis (Russell et al. 2012; Stalder et al. 2017).

We examine these relationships within the context of Mexican-origin adolescents from low-income immigrant families in Texas for three main reasons. First, by 2060, one in three youth in the United States will be of Latino background, with Mexican-origin individuals being the largest and fastest growing subgroup (Noe-Bustamante et al. 2023). Second, Texas, with the second-highest proportion of Mexican-origin families in the United States after California (Noe-Bustamante et al. 2023), provides valuable insights into how neighborhood contexts may influence the stress physiology of Mexican-origin youth. Texas, with neighborhoods ranging from 0% to 100% Latino and immigrant concentrations, offers an ideal setting to explore this impact. This variation is crucial because regions with limited Latino concentration may have diverse racial–ethnic compositions rather than homogeneity (Browning, Dirlam, and Boettner 2016; White, Zeiders, and Safa 2018), an important aspect of neighborhood-level social organization (Sampson et al. 1997). Unlike California, Texas has a sociopolitical landscape characterized by more hostile anti-immigrant laws and sentiments, raising questions about how such context may affect the significance and function of co-ethnic neighborhoods among Mexican-origin youth. Third, by employing a racially and ethnically homogeneous research design, we recognize the distinct experiences of various racial and ethnic neighborhoods (e.g., Black, Asian, or Latino concentration) by both in- and out-group members, as highlighted by other researchers (e.g., Roosa et al. 2008; White, Zeiders, and Safa 2018). In the following sections, we briefly describe different theoretical frameworks that guide our hypotheses and review the relevant background literature.

### 1.1 ∣ Mainstream Perspectives of Neighborhood Effect: Allostatic Load and Social Disorganization Frameworks

Higher concentrations of neighborhood socioeconomic disadvantage typically endure higher levels of neighborhood disorder across various aspects (Massey and Denton 1993). These areas frequently exhibit elevated crime rates and physical disorder, manifested through activities, such as graffiti, pollution, dumping, and the presence of abandoned or poorly maintained buildings (Anderson and Oncken 2020). From an allostatic load perspective (Massey 2004; McEwen 2000), chronic exposure to neighborhood disorder can pose psychosocial and physical threats to individuals and can lead to a chronic physiological stress response of the HPA axis. Chronic stress response, as manifested through elevated cortisol levels, can in turn damage health in the long run (McEwen 2000). Relatedly, social disorganization theory posits that neighborhood concentrated poverty (and resource deprivation) would undermine neighbors’ abilities to develop informal social control (e.g., neighborhood monitoring; Sampson, Raudenbush, and Earls 1997). This, in turn, is expected to undermine the health and well-being of residents. Collectively, based on the allostatic load and social disorganization frameworks, we would expect that Mexican-origin youth living in neighborhoods with higher (relative to lower) socioeconomic disadvantage may exhibit higher levels of HCC due to neighborhood disorders, whereas those living in neighborhoods with higher (relative to lower) affluence may exhibit lower levels of HCC as neighborhood affluence attracts health-related resources (Clarke et al. 2014).

### 1.2 ∣ Cultural-Developmental Neighborhood Perspectives: Person–Environment Fit Framework

Mainstream neighborhood effects research offers insights into adolescent health but often neglects the role of racial–ethnic, cultural, and social identities in shaping these impacts (Witherspoon et al. 2023). The person–environment fit framework challenges the idea of adolescents as passive recipients of neighborhood influence (Roosa et al. 2009), proposing that adaptation depends on the alignment among contextual features, individual traits, and identities. The better the alignment, the more favorable the adjustment (Caplan 1987; Roosa et al. 2009). Conversely, poor adjustment, indicated by higher stress physiology in our study, is more likely with greater mismatch, highlighting the need to evaluate both individual and contextual characteristics (Caplan 1987). We consider adolescents’ ethnic and economic backgrounds as individual factors interacting with neighborhood characteristics.

Mexican-origin youth residing in neighborhood with higher co-ethnic density may feel more comfortable and experience less stress, given that this environmental context “fit” with their ethnic background. Co-ethnic neighborhoods are thought to act as “ethnic enclaves” that provide sociocultural symbols and resources tailored to the needs and preferences of the community (Logan et al. 2002; Pasco, White, and Seaton 2021; Portes and Manning 2019; Yoshikawa 2011). Studies suggest that neighborhoods with higher Latino concentration strengthen Mexican heritage identification among adolescents, correlating with increased school attachment, greater racial–ethnic identity exploration, and reduced peer discrimination in middle adolescence, ultimately leading to fewer problems later (White, Kho, and Nair 2021; White, Zeiders, and Safa 2018). Additionally, Latino concentration acts as a protective factor against early pubertal timing’s negative effects on Mexican-origin boys (White et al. 2013). Collectively, we would expect Mexican-origin youth residing in neighborhoods with higher Hispanic-origin residents (as a proxy of co-ethnic density) to exhibit lower HCC levels, given that youth who experience high levels of sociocultural resources and support tend to experience less stress (Campos, Yim, and Busse 2018).

In contrast, person–environment misfit may arise when Mexican-origin youth in more racially and ethnically integrated neighborhoods feel out of place due to a lack of commonalities with their neighbors. We specifically examine the impact of predominantly non-Hispanic White neighborhoods on Mexican-origin youth for two reasons. First, non-Hispanic Whites often shape mainstream culture; thus, their presence in a neighborhood reflects prevailing cultural norms (Caplan 1987). Second, evidence suggests that Mexican-origin adolescents in such neighborhoods may experience increased externalizing symptoms, potentially due to challenges with cultural adaptation and social integration (White, Kho, and Nair 2021). Additionally, person–environment misfit can stem from neighborhood socioeconomic disparities. Mexican-origin adolescents from low-income families residing in affluent neighborhoods may experience stress due to financial burdens and limited job availability. For example, moving to more affluent neighborhoods has been associated with higher levels of maladaptive outcomes, such as lower school performance and increased substance use among youth from low-income Black and Latino families (Fauth et al. 2007). However, affluent neighbors might provide benefits to low-income youth (Clarke et al. 2014), leaving the physiological impact unclear. Moreover, prior studies often failed to differentiate between the effects of neighborhood SES and racial–ethnic composition, despite their high correlation. Consequently, the unique role of non-Hispanic White neighborhoods independent of neighborhood affluence, and vice versa, remains unclear. Nevertheless, based on the person–environment fit framework, we anticipate that Mexican-origin adolescents from low-income immigrant families in neighborhoods with higher proportions of non-Hispanic White residents and/or higher affluence may exhibit higher HCC levels, indicating a potential misfit.

### 1.3 ∣ HCC as Chronic Physiological Stress Response

Glucocorticoids (i.e., cortisol in humans), the end-product of HPA activation, serve as indicators of stress responses and play roles in modulation and preparative actions (Gunnar and Quevedo 2007; Sapolsky, Romero, and Munck 2000). Unlike salivary or urine cortisol, which is often used to assess acute stress responses over a relatively short timeframe (e.g., a few days), hair cortisol represents the accumulation of unbound, free (active) circulating cortisol incorporated into growing hair over an extended period (e.g., Russell et al. 2012; Stalder et al. 2017). As such, HCC is increasingly recognized as a potential indicator for retrospectively tracking cumulative physiological stress responses (Liu and Doan 2019; Russell et al. 2012). For example, Pereg et al. (2011) investigated the role of chronic stress as measured by hair cortisol 3 months *before* the development of a heart attack (acute myocardial infarction; AMI) and found that hair cortisol was the strongest predictor (followed by BMI) of AMI, even after accounting for age, lipid status, smoking, and other predictors. This finding thus suggests that HCC could be one of many potential biomarkers of understanding health and health disparities.

However, it is important to also acknowledge that, similar to other cross-sectional measures that can only provide a “snapshot” of an individual, HCC can only objectively assess chronic stress level over ~3 months (1 cm of hair reflects 1 month of cortisol secretion). Other limitations in employing a single value of HCC include individual variability in hair growth rates and the difficulty in pinpointing specific stressors or acute events (Russell et al. 2012; Stalder et al. 2012). Nevertheless, unlike cortisol extracted from urine and saliva, two independent studies have demonstrated that HCC showed high test–retest associations between repeated HCC assessments over 1-year period and three times at 2-month intervals, respectively. This suggests a high intraindividual stability of HCC (Stalder et al. 2012). On the other hand, meta-analytic evidence has revealed that, despite HCC being a reliable biomarker of chronic stress, self-reports of perceived stress often do not align with HCC levels (Stalder et al. 2017), even when a large sample of participants is used (Planert et al. 2023). Although the underlying reasons remain unclear, previous studies may have relied solely on one-time retrospective assessments of perceived stress, which can be prone to subjective bias and may not accurately reflect long-term cortisol secretion measured through HCC (Stalder and Kirschbaum 2012). Therefore, we employ HCC as a direct biological assessment of adolescent health and stress, as opposed to relying on subjective self-report measures of stress.

### 1.4 ∣ The Role of Household and Neighborhood Contexts on HCC

The associations between SES and HCC are equivocal (Bryson et al. 2021). Moreover, these studies have primarily focused on young children primarily from middle-class and non-Hispanic White backgrounds in the US (e.g., Tarullo et al. 2020) or from non-US geographical contexts (e.g., Bryson et al. 2019; Rippe et al. 2016; Vaghri et al. 2013). Among existing studies that have linked HCC with household SES, lower parental education (Tarullo et al. 2020; Ursache et al. 2017; Vaghri et al. 2013) and household income (Rippe et al. 2016) were associated with higher HCC among typically developing preschool- and early school-aged children. Other studies have found no association between household SES (income and education) and HCC among children (see Bryson et al. 2021 for a review).

Among studies that have linked HCC with neighborhood SES, higher housing problems (e.g., overcrowding, mold) were associated with lower HCC among 2-year-old children, even after controlling for household SES. However, low-tenure housing (e.g., public rental) was associated with higher child HCC (Bryson et al. 2019). Although different theoretical models have been proposed, empirical studies on how neighborhood SES and racial–ethnic composition influence stress physiology (as indexed by HCC) among Mexican-origin youth are limited.

### 1.5 ∣ The Current Study

Drawing on various prominent theories (i.e., allostatic load, social disorganization, and person–environment fit frameworks), we empirically test the associations between neighborhood SES, neighborhood racial–ethnic and immigrant structure, and HCC among Mexican-origin adolescents from low-income immigrant families. Neighborhood SES is assessed by two indices: (1) neighborhood disadvantage and (2) neighborhood affluence. Neighborhood disadvantage is operationalized as a factor score based on four census indicators: the proportion of female-headed families with children, the proportion of households with public assistance income or food stamps, the proportion of families with income below the federal poverty level, and the proportion of the population age 16+ unemployed within a neighborhood. Neighborhood affluence is operationalized as a factor score based on three census indicators, including the proportion of households with income greater than $75k, the proportion of the population age 16+ employed in professional or managerial occupations, and the proportion of adults with a bachelor’s degree or higher within a neighborhood. Neighborhood racial-ethnic and immigrant structure is assessed by three indices: (1) proportion of non-Hispanic White residents, (2) proportion of Hispanic-origin residents, and (3) proportion of foreign-born residents. These indexes are continuous measures assessing variabilities across neighborhoods in terms of the specific residents in the neighborhood. For example, the index of the proportion of Hispanic-origin residents assesses variabilities across neighborhoods in terms of the percentage of Hispanic-origin residents. Higher values reflect neighborhood with high proportions of Hispanic-origin residents, whereas lower values reflect neighborhood with higher non-Hispanic-origin residents.

#### 1.5.1 ∣ Aims/Hypotheses

To better contextualize the neighborhood environment in which our sample of Mexican-origin adolescents from low-income immigrant families resides, we first examined the associations among neighborhood SES (i.e., neighborhood disadvantage and affluence) and neighborhood racial–ethnic and immigrant structure (i.e., the proportion of non-Hispanic White residents, the proportion of Hispanic-origin residents, and the proportion of foreign-born residents) at the neighborhood level. Based on the effects of racial–ethnic residential segregation (Acevedo-Garcia et al. 2013; Williams and Collins 2001), we hypothesized that neighborhoods with a higher proportion of Hispanic-origin or foreign-born residents would be associated with higher neighborhood disadvantage and lower neighborhood affluence. In contrast, neighborhoods with a higher proportion of non-Hispanic White residents would be associated with higher neighborhood affluence and lower socioeconomic disadvantage.

Second, using a multilevel modeling approach to account for the nesting effect of households within neighborhoods, we examined the association between household characteristics (i.e., parental education and income) and neighborhood SES (i.e., neighborhood socioeconomic disadvantage and affluence) and HCC among Mexican-origin adolescents. Covariates included adolescents’ sex, age, and nativity. We hypothesized that neighborhood socioeconomic disadvantage and affluence would be associated with HCC, above and beyond the effect of parental education and income, given that the neighborhood is a salient aspect of adolescent development. Based on the allostatic load and social disorganization theories, we would expect that higher neighborhood socioeconomic disadvantage would be associated with higher HCC among youth. However, it is unclear, as some evidence suggests only a weak association between neighborhood socioeconomic disadvantage and stressful experiences among Mexican-origin youth (Roosa et al. 2010). Based on the person–environment fit theory, we would expect that higher neighborhood affluence would be associated with higher HCC among Mexican-origin adolescents from low-income families, although it remains unclear given the available evidence.

Third, using a multilevel modeling approach and the same covariates, we examined the associations among neighborhood racial–ethnic and immigrant structure (i.e., the proportion of non-Hispanic White, the proportion of Hispanic-origin, and the proportion of foreign-born residents) within a neighborhood and HCC among Mexican-origin adolescents from low-income immigrant families, after accounting for household SES. Drawing on the person-environment fit framework, we would expect that Mexican-origin adolescents living in neighborhoods with a higher (vs. lower) proportion of Hispanic-origin residents would exhibit lower HCC, whereas youth living in neighborhoods with a higher (vs. lower) proportion of non-Hispanic White residents would exhibit higher HCC (White, Kho, and Nair 2021).

Finally, given that neighborhood SES and racial–ethnic and immigrant structure are often correlated due to segregation, we conducted sensitivity to disentangle the distinct effects of neighborhood SES and neighborhood racial–ethnic and immigrant structure while controlling for each other within the same model.

This study offers three key implications. First, understanding how neighborhood variations affect adolescents’ stress physiology (as indexed by HCC) offers insights beyond subjective reports of stress and behaviors. Despite being a reliable biomarker, self-reported stress often does not align with HCC levels, even in large samples (Planert et al. 2023; Stalder et al. 2017). Moreover, research suggests that although a subset of upwardly mobile African American youth who were raised in impoverished neighborhoods show resilience academically and behaviorally (e.g., excelling in school and showing lower rates of drug use), this resilience may be “skin deep” and come at a physiological cost, as evidenced by elevated allostatic load (e.g., increased cortisol levels; Chen et al. 2015). Thus, utilizing HCC as a noninvasive method to assess chronic stress responses holds promise for understanding neighborhood effects on adolescent physiological health. Second, our study represents a fundamental step toward understanding the biological pathways of health disparities often experienced by racial–ethnic minorities (Massey 2004; Williams and Collins 2001). Third, our study findings have potential implications for informing housing interventions and policies.

## 2 ∣ Methods

### 2.1 ∣ Participants and Procedures

Data were collected from a larger longitudinal study conducted among low-income Mexican-origin immigrant families recruited in central Texas between 2012 and 2020 through convenience sampling. The study was conducted in three waves: Wave 1 (2012–2015), Wave 2 (2013–2016), and Wave 3 (2017–2020). Families were eligible for participation in the larger study if they had at least one child in middle school who could act as a translator or interpreter for their parents between Spanish and English. The data used in the current study include adolescents’ and mothers’ responses obtained during home visits in Wave 3 (*N* = 334), as well as hair cortisol data from adolescents (*N* = 301) collected either before or during the interviews. Adolescents with hair cortisol values that fell below or exceeded three standard deviations from the mean were considered outliers and were subsequently removed from the analysis (*n* = 4), following recommendations (Enlow et al. 2019). In total, the current study included 297 adolescents (mean age = 17.61, SD = 0.93) and their mothers (*N* = 292; mean age = 43.26, SD = 5.74). Of the adolescent participants, 161 were females (54.20%), 136 were males (45.80%), and the majority were born in the United States (76.10%). The vast majority of mothers were born in Mexico (98.90%), and their average length of permanent residence in the United States was 19.85 years. The median and mode of mothers’ education level were at the middle school or junior high school level. The median and mean household family income for participants fell within the range of $30,001–$40,000.

During the home-visit interview, bilingual research assistants read questions aloud to participants in their preferred language (Spanish or English) and recorded their responses by inputting them into a laptop computer. All questions were prepared in both Spanish and English, and research assistants translated the original English questions into Spanish and back-translated them into English to validate the translations. Each participating family received $90 as compensation for their involvement in the interview.

Hair samples were collected by trained research assistants during home visits. Research assistants carefully cut hair strands from adolescents using sterilized scissors, as close to the scalp as possible, from the posterior vertex position. Research assistants were instructed to obtain approximately 100 strands of hair, with each strand being at least 3 cm in length. Hair samples were placed on aluminum foils, and the end of the hair closer to the scalp was marked for strands longer than 3 cm. Each participating family received $20 for the hair collection. The hair samples were stored at room temperature and later analyzed at the Kirschbaum Endocrine Laboratory at the Technical University Dresden in Germany.

### 2.2 ∣ Measures

#### 2.2.1 ∣ Adolescent Hair Cortisol Extraction

The hair strands were lined up and cut into a 3 cm segment in the laboratory, corresponding to hair cortisol in approximately the prior 3 months. For the washing of hair and cortisol extraction, the protocol of Davenport et al. (2006) was employed. In brief, each hair segment was put into a 10 mL glass container, then 2.5 mL isopropanol was added, and the tube was gently mixed on an overhead rotator for 3 min. After decanting, the wash cycle was repeated twice. Then the hair samples were allowed to dry for at least 12 h. Next, the hair segment was weighed, and 7.5 mg were transferred into a 2 mL cryovial. of Pure methanol of 1.5 mL was added, and the cortisol extraction was performed for 18 h. Samples were then spun in a microcentrifuge at 10,000 rpm for 2 min, and 1 mL of the clear supernatant was transferred into a new 2 mL glass vial. The alcohol was evaporated at 50° C under a constant stream of nitrogen until the samples were completely dried. Finally, 0.4 mL of water was added, and the tube vortexed for 15 s. Fifty microliters were removed from the vial and used for cortisol determination with commercially available immunoassays with chemiluminescence detection (CLIA, IBL). The hair extract was injected two times, and chromatograms evaluated them individually. The HCC value is calculated based on the average of the two duplicates. The intra- and inter-assay coefficients of variance (CV) for this assay are 9.9% and 12.7%, respectively. The CV computations are based on values greater than the levels of detection, which are 0.94 pg/mg.

#### 2.2.2 ∣ Household SES

*Family income* was measured using a single item that inquired about annual family income (i.e., think of all the income from persons in your family who live in the same house with you. Consider all kinds of income including wages earned from jobs, alimony, unemployment compensation, and government assistance. What was your family’s income from all?). Mothers answered this item on a scale from 0 ($10,000 or under $10,000) to 11 ($110,001 or more).

*Mother education* was measured using a single item that asked about the highest education level (i.e., What is the highest level of education you have completed?). Mothers answered this item on a scale ranging from 1 (*no formal schooling*) to 11 (*finished graduate degree*).

#### 2.2.3 ∣ Neighborhood Contexts

We derived theoretically based measures of neighborhood SES and neighborhood racial–ethnic and immigrant structure from the National Neighborhood Data Archive (NaNDA; https://nanda.isr.umich.edu/). Data on neighborhood demographics, including proportions per ZIP Code Tabulation Areas (ZCTAs), were sourced from the US Census Bureau’s American Community Survey (ACS), representing the SES and demographics of each census tract from the years 2013 to 2017. The ACS 5-year estimates are commonly used for capturing neighborhood characteristics as they enhance the statistical reliability of data for less populated areas and small population subgroups. Moreover, these estimates are available for all geographic areas down to the ZCTA level. Geospatial information for each ZCTA was obtained from the TIGER/Line shapefiles, released in 2019 by the US Census Bureau, to ensure consistency with similar datasets from NaNDA (Robert Melendez et al. 2020). We geocoded each family’s residential address using the family’s ZIP code during Wave 2 (2013–2016), which is aligned with the 2013–2017 ACS estimates, in order to examine the longitudinal impact of neighborhood contexts on adolescents’ HPA axis. Neighborhood indices were integrated into our study through a ZIP code to ZCTA crosswalk.

#### 2.2.4 ∣ Neighborhood SES

Neighborhood SES factors were generated using principal factor analysis with orthogonal varimax rotation on seven census indicators (log-transformed to correct positive skew), following the approach outlined by Morenoff et al. (2007). *Neighborhood disadvantage* is represented as a factor score based on four census indicators within a neighborhood defined at the ZCTA level. These indicators include the proportion of female-headed families with children, the proportion of households receiving public assistance income or food stamps, the proportion of families with income below the federal poverty level, and the proportion of the population aged 16 and older who are unemployed. The scores range from 0 to 1.0. *Neighborhood affluence* is a factor score based on three census indicators within a neighborhood, as defined at the ZCTA level. These indicators comprise the proportion of households with income greater than $75k, the proportion of the population aged 16 and older employed in professional or managerial occupations, and the proportion of adults holding a bachelor’s degree or higher. The scores also range from 0 to 1.0.

#### 2.2.5 ∣ Neighborhood Racial–Ethnic and Immigrant Structure

Neighborhood racial–ethnic and immigrant structure was assessed using three indices at the ZCTA level: (a) the proportion of non-Hispanic White residents, (b) the proportion of Hispanic-origin residents, and (c) the proportion of residents who are foreign-born. Higher values of these indices indicate a greater proportion of specific residents residing in the neighborhood.

### 2.3 ∣ Analysis Plan

Data analyses were conducted in SPSS 22.0 and Mplus 8.3 (Byrne 2013) in two steps. First, we performed descriptive analysis and calculated Pearson product–moment correlations among study variables using SPSS 22.0. Second, considering that families were nested within neighborhoods, we conducted multilevel analyses to investigate the associations between family and neighborhood indices and adolescents’ hair cortisol levels. Each neighborhood index (i.e., neighborhood disadvantage, neighborhood affluence, proportion of non-Hispanic White residents, proportion of Latino residents, and proportion of residents who are foreign-born) was modeled separately to prevent multicollinearity. We assessed the missingness of study variables using Little’s missing completely at random test, which indicated that the missing data were completely at random (*χ*^2^(8) = 15.160, *p* = 0.056). Consequently, we included families that had complete data on all study variables in the multilevel analyses, resulting in a sample size of 222 families.

## 3 ∣ Results

### 3.1 ∣ Descriptive Statistics and Pearson Correlation

Table 1 shows the correlation and descriptive information of study variables at the family level. Higher neighborhood affluence was associated with higher levels of adolescents’ HCC. Moreover, living in neighborhood with a higher proportion of Hispanic-origin residents was associated with lower HCC, whereas living in neighborhood with a higher proportion of non-Hispanic White residents was associated with higher levels of HCC among adolescents.

Question 1. What are the associations among neighborhood SES and neighborhood racial–ethnic and immigrant structuring?
  As indicated in Table 2, when examining the neighborhood level, neighborhoods with a higher proportion of non-Hispanic White residents were strongly associated with higher levels of neighborhood affluence and lower levels of neighborhood disadvantage. Conversely, neighborhoods with a higher proportion of Latino and foreign-born residents were strongly linked to higher neighborhood disadvantage and lower neighborhood affluence.

**FIGURE 1 ∣:**
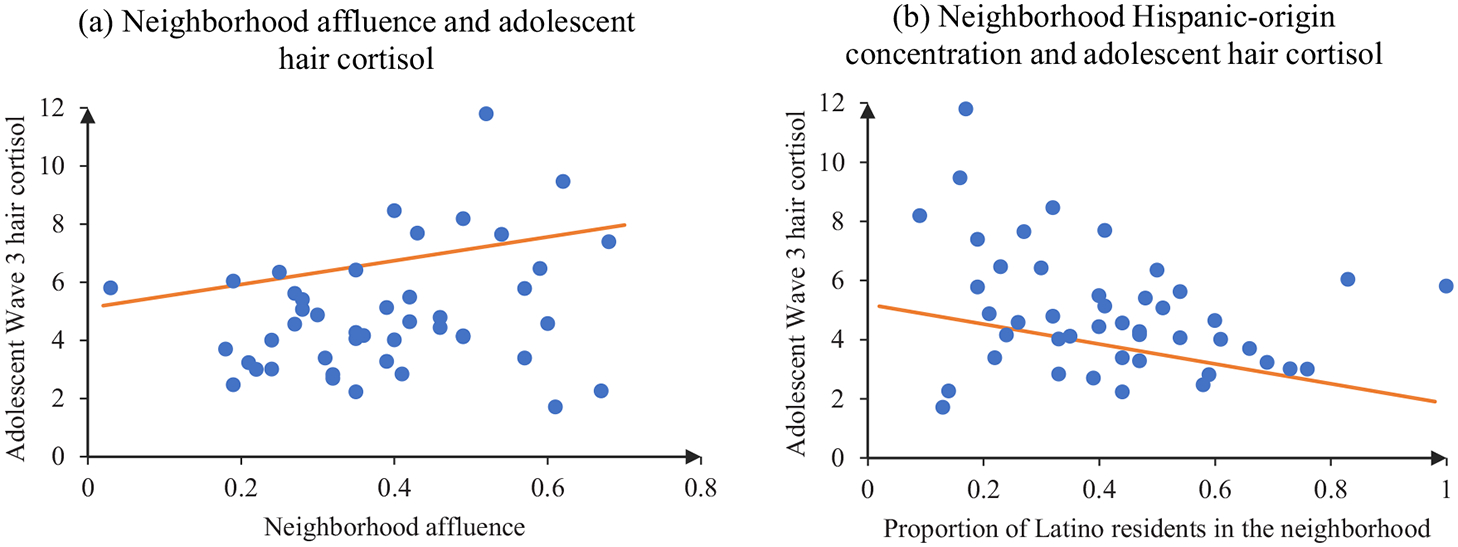
Scatter plots of (a) neighborhood affluence, (b) proportion of Hispanic-origin residents, and adolescent Wave 3 hair cortisol at neighborhood-level. The orange solid line shows the estimated regression coefficient from the multilevel results.^1^ *Y*-axis shows the aggregated adolescent hair cortisol at the neighborhood level.

Question 2. What are the associations between household and neighborhood SES and HCC among Mexican-origin adolescents from low-income immigrant families?
  Multilevel modeling revealed that residing in a more affluent neighborhood was associated with *elevated* HCC levels (*b* = 4.073, *p* < 0.05; see Table 3 and Figure 1a) in Mexican-origin adolescents from low-income immigrant families. This association held even after controlling for household SES, as well as the effects of adolescents’ sex, nativity, and age. Additionally, male adolescents exhibited higher HCC levels than their female counterparts, whereas no other significant associations were observed.

Question 3. What are the associations among neighborhood racial–ethnic and immigrant structuring andHCC among Mexican-origin adolescents from low-income immigrant families?
  Multilevel modeling revealed that residing in a neighborhood with a higher proportion of Hispanic-origin residents was significantly associated with lower HCC levels (*b* = −3.362, *p* < 0.001; see Table 4 and Figure 1b) among Mexican-origin adolescents from low-income immigrant families. This association remained significant after controlling for household SES, adolescents’ sex, nativity, and age. Additionally, living in a neighborhood with a higher proportion of foreign-born residents was marginally associated with lower adolescent HCC levels (*b* = −4.204, *p* = 0.055).

### 3.2 ∣ Sensitivity Analyses

We conducted sensitivity analyses to disentangle the distinct effects of neighborhood SES (i.e., neighborhood affluence) and neighborhood racial–ethnic structure (i.e., proportional of Hispanic-origin residents) on HCCs while controlling for each other within the same model. In the model, we correlated the residuals of neighborhood affluence and the proportion of Hispanic-origin residents to account for their correlations.

The results revealed that a neighborhood with a higher proportion of Hispanic-origin residents was marginally associated with *lower* HCC among adolescents (*b* = −3.625, SE = 2.089, *p* = 0.083) after accounting for neighborhood affluence. However, when we controlled for the proportion of Hispanic-origin residents, neighborhood affluence was no longer associated with adolescents’ HCC (*b* = −0.416, SE = 3.298, *p* = 0.900).

These sensitivity analyses suggest that the observed association between higher neighborhood affluence and elevated HCC in adolescents, as indicated in the main results, may be attributed to, or explained by, the negative association between neighborhood affluence and the proportion of Hispanic-origin residents. In other words, the reason why adolescents living in more affluent neighborhoods had higher HCC levels may be linked to the fact that those in affluent neighborhoods had lower proportions of Hispanic-origin residents, which, in turn, was associated with higher HCC levels in Mexican-origin adolescents from immigrant families.

## 4 ∣ Discussion

Our objective in the current study was to explore the connections between neighborhood factors, including SES and ethnic–racial and immigrant composition, and adolescents’ HCC—a physiological marker of chronic stress response. Our findings indicated that neighborhoods with a higher proportion of Hispanic-origin or foreign-born residents were associated with greater neighborhood disadvantage and lower neighborhood affluence. Conversely, neighborhoods with a higher proportion of non-Hispanic White residents and domestic-born residents were linked to reduced disadvantage and increased neighborhood affluence. These results align with the extensive literature on how US sociopolitical policies and racial residential segregation have disproportionately funneled families of color into more socioeconomically disadvantaged neighborhoods (Massey 2001). These conditions potentially have intergenerational consequences for subsequent generations, as supported by the perpetuation theory (Goldsmith 2016). Critically, residing in a more affluent neighborhood was associated with *elevated* HCC levels, whereas residing in a neighborhood with a higher proportion of Hispanic-origin residents was significantly associated with lower HCC levels.

### 4.1 ∣ Neighborhood Racial–Ethnic and Immigrant Structure and HCC

In line with our hypotheses, we discovered that Mexican-origin adolescents residing in neighborhoods with a higher proportion of Hispanic-origin (but not foreign-born) residents exhibited lower levels of HCC. This association held even after controlling for household SES as well as the effects of adolescents’ sex, nativity, and age. Prominent theories (e.g., ethnic enclave and person-environment fit theory) propose that neighborhoods with high co-ethnic density can promote community trust, mutual support, and informal social control, leading to various positive outcomes for individual residents (Browning, Dirlam, and Boettner 2016; Lee and Martinez 2002; Logan et al. 2002; Martinez and Polo 2018; Pasco, White, Iida, et al. 2021; Rumbaut and Portes 2001; White, Kho, and Nair 2021; White, Zeiders, and Safa 2018; Witherspoon et al. 2023). Indeed, Mexican-origin adolescents living in neighborhoods with a higher concentration of co-ethnic (Hispanic-origin) members tend to experience fewer externalizing and internalizing symptoms (Basáñez et al. 2013; Martinez and Polo 2018; Portes and Manning 2019; White, Zeiders, and Safa 2018).

Moreover, residing in neighborhood with higher co-ethnic density can reinforce the values that are important to the Mexican-origin community, such as ethnic identity and family values. This, in turn, contributes to greater acceptance, adoption, and internalization of core cultural beliefs, acting as protective factors against the influence of stress (Martinez and Polo 2018; Pasco, White, Iida, et al. 2021). For example, a study found that Mexican-origin mothers’ perception of neighborhood social and cultural cohesion increased maternal cultural socialization, which in turn promoted ethnic–racial identity processes and content among Mexican-origin adolescents (Pasco, White, Iida, et al. 2021). Additionally, it is plausible that adolescents experience fewer incidents of racism and discrimination when living in neighborhoods with a higher concentration of co-ethnics (White, Kho, and Nair 2021), resulting in reduced stress. Future studies, however, should further examine the proximal mechanisms, such as perceived social support, cohesion, cultural socialization, and reduced discrimination, that link neighborhood with higher Hispanic-origin concentration to lower HCC among Mexican-origin adolescents.

### 4.2 ∣ Neighborhood SES and HCC

We observed that Mexican-origin adolescents in neighborhoods with higher affluence displayed elevated HCC, which typically indicates a heightened physiological stress response. This finding is intriguing since affluence is usually associated with better adolescent health (Leventhal & Dupéré 2019). Although further research is needed to validate this, we propose several explanations for this observed finding.

First, supporting the person–environment fit theory, Mexican-origin youths from low-income families in affluent neighborhoods may feel more isolated, as affluence can accentuate the distinctions between the “haves” and “have-nots.” For instance, they may encounter disparities in access to resources, education, or job opportunities, which can exacerbate feelings of exclusion and disconnection. This heightened awareness can potentially activate the body’s stress response system and may contribute to increased cortisol levels (Cantave et al. 2023).

Second, although we did not find direct association between neighborhood non-Hispanic Whiteness and HCC, affluent neighborhoods tend to be predominantly White (see Table 1). Mexican-origin adolescents from low-income immigrant families may feel unwelcome in such neighborhoods due to hypervisibility, presumed criminality, and policing, as suggested by previous research (Posey-Maddox 2017). Additionally, prior studies have found that moving to whiter neighborhoods predicted *increased* externalizing problems among Mexican-origin youth (White, Kho, and Nair 2021). Notably, neighborhood affluence was no longer associated with cortisol levels after controlling for co-ethnic concentration in our sensitivity analyses. This suggests that racial–ethnic and immigrant structuring of a neighborhood has a more significant impact on HCC than neighborhood affluence.

Third, our findings may be partially explained by the skin-deep resilience theory, which suggests that upward social mobility may come with a physiological cost, as evidenced by elevated allostatic load (e.g., increased cortisol levels; Chen et al. 2015). This theory has only been empirically tested among samples of African American young adults. Our study did not directly assess social mobility or achievement outcomes. Hence, future studies are needed to fully understand whether the observed findings could be explained by this theory.

We did not find any associations between neighborhood disadvantage and adolescents’ HCC, despite the assumption that high disadvantage neighborhoods constitute chronic stressors. We also found no evidence of a connection between household SES and HCC. Although the present findings contradict frameworks such as allostatic load and social disorganization theory—which posit that neighborhood socioeconomic disadvantages undermine the health and well-being of residents (Sampson 2002)—they align with previous research indicating that Latino concentration, rather than concentrated poverty, predicts better mental health among Mexican-origin adolescents (White, Zeiders, and Safa 2018). Indeed, one study found that there was only weak association between neighborhood socioeconomic disadvantage and stressful experiences among Mexican-origin youth (Roosa et al. 2010). Relatedly, although we did not set out to test the competing narratives of segregation and diversity, our findings here provide some hints that perhaps, in some nuanced circumstances, segregation may work when there is community support and resources to capitalize upon.

Collectively, our findings underscore the importance of considering neighborhood contextual factors and the multifaceted ecological contexts in which youth live to understand their health outcomes. To promote Mexican-origin youth’s well-being and better understand the links with their biological and physical health, further research is needed to determine how neighborhood affluence, disadvantage, and household SES independently and collectively contribute to adolescents’ biological health.

### 4.3 ∣ Implication and Future Directions

Our findings have important implications and suggest directions for future research. We point out that our study does not aim to critique US social housing policy or assess specific housing programs. Our cross-sectional study’s limitations prevented us from examining changes in residential neighborhoods (e.g., mobility). Nevertheless, our study likely suggests that relocating low-income racial–ethnic minority families to more affluent neighborhoods without understanding their racial–ethnic structuring and providing sociocultural support could yield unintended consequences (e.g., Fauth et al. 2007). Given that mobility theories are often overlooked, future research should consider mobility effects to better understand how neighborhood factors, such as affluence, disadvantage, and ethnic concentration, impact the well-being of Mexican-origin families.

Additionally, adhering to the immigrant revitalization approach, enhancing neighborhood community infrastructures can serve as a protective factor for racial–ethnic minority and immigrant-origin youth against the effects of stress. Future research should delve into potential mediators, like social capital, support, community cohesion, and discrimination, to explain these outcomes. This can be done through mixed-method approaches and ethnographic interviews (e.g., Pasco and White 2020) to better contextualize their experiences in affluent neighborhood. Furthermore, although the exact mechanisms behind the association between neighborhood contexts and physiological responses remain unclear, there is an opportunity for prevention and intervention efforts to improve the well-being of Mexican-origin youth. Local and national agencies can provide social and community support to low-income immigrant-origin youth in affluent neighborhoods, promoting a sense of belonging through cultural awareness programs and community-building initiatives.

Lastly, individual heterogeneity in stress exposure features, such as the chronicity and developmental timing of neighborhood conditions, should be considered when examining the stress response of the HPA axis.

### 4.4 ∣ Strengths and Limitations

This study has several strengths. First, it separates neighborhood SES into disadvantage and affluence, examining both aspects in one study rather than relying on an overarching neighborhood SES construct. Second, it explores how neighborhood racial–ethnic and immigrant structuring influences Mexican-origin adolescents’ HPA axis using multilevel modeling.

However, there are notable limitations. First, our methods assume equal exposure for all adolescents in a neighborhood, and we did not include measures of adolescents’ subjective experiences, such as sense of isolation and belongingness and neighborhood cohesion. Subjective perceptions of the demographics of their neighborhoods may serve as psychological proxies that explain the link between neighborhood contexts and HCC levels. Considering that policy interventions targeting subjective experiences (e.g., feelings of isolation and belonging) could potentially have a positive impact on addressing Mexican-origin families’ health concerns within affluent neighborhoods, it was thus a limitation that these measures were not included in our study. Additionally, future studies could employ global positioning systems and ecological momentary assessment to capture these experiences more accurately. Second, due to resource constraints, our study only measured neighborhood indicators at the ZCTA level. Although research has shown similarities between ZCTA and census-track level measures in predicting health outcomes (e.g., Berkowitz et al. 2015; Goetschius et al. 2023; Thomas et al. 2006), there may be variation in findings based on granularity (Pasco, White, and Seaton 2021; Tian et al. 2010). Future research should investigate this further. Nevertheless, neighborhood indicators at ZCTA and tract level both provide useful information. In fact, policy efforts are generally aimed at larger areas, so using more granular measures may be less useful for identifying targets for intervention. Third, the study used a homogeneous sample of Mexican-origin adolescents in a specific region, limiting generalizability to other racial–ethnic or socioeconomic groups. In relation, the nature of our dataset did not include other neighborhood data that specified other racial/ethnic groups in the neighborhood (e.g., predominantly Black or Asian neighborhoods), which precludes the examination of the effect of living in these neighborhoods (e.g., predominantly Black or Asian neighborhoods) among Mexican-origin youth. Future work should consider diverse youth samples from other regions of the country to explore how neighborhood factors affect health outcomes. Fourth, the study’s reliance on the ACS with 5 years of data may not capture changes in youth’s living conditions within that period. Timing effects and causal relations should be examined in future research. Fifth, cortisol levels are linked to pubertal development. Despite including adolescent age as a covariate in our study, this may provide an imperfect assessment of pubertal development, particularly within the limited age range of the current study. The absence of other variables related to pubertal development in our study represents a methodological limitation. Finally, the study was constrained in that we solely measured the physiological response to stress through adolescents’ hair cortisol levels. Consequently, our findings likely pertain to associations with adolescents’ HPA axis activation, leaving the relationship between individual differences in cortisol and adolescents’ functional and behavioral outcomes unclear (Hackman, Betancourt, et al. 2012).

## 5 ∣ Conclusion

Addressing the physiological health and overall well-being of adolescents requires a multilevel approach that encompasses upstream macrosocial causes, such as neighborhood contexts. It also involves investigating the biological mechanisms at play during adolescent development, a period when processes may be more amenable to promotion, intervention, or prevention. Our findings underscore the significance of neighborhood research, as they demonstrate that neighborhood contexts, along with household and individual factors, influence adolescents’ HPA axis. Furthermore, our results shed light on the neighborhood conditions that can contribute to the thriving of Mexican-origin adolescents.

Additionally, our findings can provide valuable insights to better inform US social housing policies. They highlight the neighborhood factors that can positively impact youth’s physical health, as well as those that may pose threats. We argue that social policies, which have traditionally focused solely on neighborhood SES, need to place more emphasis on understanding the racial-ethnic and immigrant composition of their neighborhoods. Additionally, they should take sociocultural contexts and person–environment fit into consideration when understanding how neighborhoods influence adolescents’ stress physiology. Our study underscores the need to carefully consider the subjective experiences and the relevant social support needed to understand the complexity of this process. However, it is important to recognize the potential benefits that can be realized to promote adolescent growth, particularly when they live in neighborhoods with strong cultural and co-ethnic community support.

## Figures and Tables

**TABLE 1 ∣:** Family-level correlation and descriptive information of study variables.

	1	2	3	4	5	6	7	8	9	10	11
1. Sex (0 = male, 1 = female)	—										
2. Nativity (0 = United States, 1 = Mexico)	−0.024	—									
3. Adolescent age	−0.048	0.101	—								
4. Family income	−0.054	−0.079	−0.164*	—							
5. Mother education	−0.009	0.070	−0.082	0.073	—						
6. Proportion of Hispanic-origin residents	0.012	0.022	0.009	−0.045	−0.059	—					
7. Proportion of non-Hispanic White residents	0.016	−0.025	−0.030	−0.016	0.060	−0.917**	—				
8. Proportion of foreign-born residents	0.013	0.056	0.073	−0.076	−0.078	0.663**	−0.764**	—			
9. Neighborhood disadvantage (from 2013 to 2017)	−0.037	0.034	0.087	−0.101	−0.119*	0.745**	−0.781**	0.689**	—		
10. Neighborhood affluence (from 2013 to 2017)	0.030	0.000	−0.006	0.034	0.108	−0.869**	0.833**	−0.580**	−0.778**	—	
11. Adolescent hair cortisol (pg/mg)	−0.127*	−0.058	−0.024	0.033	0.028	−0.147*	0.121*	−0.096	−0.110	0.123*	—
*N*	297	297	285	245	292	279	279	279	279	279	297
*Mean* or % of 1	54%	24%	17.611	2.278	5.031	0.493	0.350	0.205	0.122	0.341	4.463
*SD*	—	—	0.934	1.549	2.283	0.171	0.176	0.087	0.045	0.122	3.210
Skewness	—	—	0.405	1.564	0.337	−0.022	0.381	0.400	0.332	0.570	2.082
Kurtosis	—	—	0.364	4.927	−0.529	−0.637	−0.628	−0.732	0.150	−0.162	5.775

Abbreviation: pg/mg, picograms per milligram.

* *p* < 0.05.

** *p* < 0.01.

**TABLE 2 ∣:** Descriptive statistics and Pearson correlations among neighborhood factors.

	1	2	3	4	5
1. Neighborhood Hispanic-origin percentage	—				
2. Neighborhood non-Hispanic White percentage	−0.914**	—			
3. Neighborhood foreign-born percentage	0.647**	−0.753**	—		
4. Neighborhood disadvantage (from 2013 to 2017)	0.726**	−0.727**	0.625**	—	
5. Neighborhood affluence (from 2013 to 2017)	−0.841**	0.748**	−0.418**	−0.717**	—
Sample average	0.49	0.35	0.20	0.12	0.34
Sample range	0.09–1.00	0.00–0.89	0.03–0.43	0.03–0.33	0.03–0.68
US national average	0.16	0.62	0.13	0.11	0.36
US national range	0–1.00	0–1.00	0–1.00	0–0.71	0–1.00

*Note*: US national average and range were retrieved from the National Neighborhood Data Archive (NaNDA): Socioeconomic Status and Demographic Characteristics of Census Tracts, United States, 2008–2017.

* *p* < 0.05.

** *p* < 0.01.

**TABLE 3 ∣:** Multi-level modeling of the effect of household and neighborhood socioeconomic status (SES) on adolescent hair cortisol concentration.

	Neighborhood disadvantage		Neighborhood affluence	
	*b (SE)*	*P*	*b (SE)*	*p*
Intercept	5.071 (0.344)	<0.001	5.117 (0.350)	<0.001
Sex	−0.805 (0.341)	0.018	−0.789 (0.337)	0.019
Nativity	−0.462 (0.504)	0.360	−0.448 (0.507)	0.377
Adolescent age	−0.021 (0.240)	0.930	−0.047 (0.233)	0.839
*Household level*				
Parental income	0.021 (0.150)	0.890	0.039 (0.150)	0.795
Mother education	0.035 (0.089)	0.696	0.031 (0.093)	0.737
*Neighborhood level*				
Neighborhood SES	−9.504 (6.392)	0.137	4.073 (2.035)	0.045
AIC	1002.538	1088.524		
Adjusted-BIC	1005.108	1091.093		
CFI	0.974	0.979		

*Note*: *N* = 222 adolescents; number of neighborhoods = 45; sex: 0—male, 1—female; nativity: 0—United States, 1—Mexico.

Abbreviations: AIC, Akaike information criterion; BIC, Bayesian information criterion.

**TABLE 4 ∣:** Multi-level modeling of the effect of household socioeconomic status (SES) and neighborhood racial–ethnic and immigrant structure on adolescent hair cortisol concentration.

	Proportion of non-Hispanic White residents		Proportion of Hispanic-origin residents		Proportion of foreign-born residents	
	*b (SE)*	*p*	*b (SE)*	*p*	*b (SE)*	*p*
Intercept	5.216 (0.444)	<0.001	5.197 (0.363)	<0.001	5.067 (0.336)	<0.001
Sex	−0.797 (0.462)	0.085	−0.747 (0.331)	0.024	−0.775 (0.337)	0.021
Nativity	−0.444 (0.521)	0.395	−0.449 (0.509)	0.377	−0.409 (0.511)	0.424
Adolescent age	−0.029 (0.255)	0.910	−0.050 (0.237)	0.834	−0.027 (0.244)	0.911
*Household level*						
Family income	0.053 (0.154)	0.730	0.028 (0.148)	0.850	0.028 (0.147)	0.850
Mother education	0.040 (0.093)	0.669	0.041 (0.094)	0.664	0.040 (0.090)	0.652
*Neighborhood level*						
Neighborhood racial–ethnic and immigrant structure	2.925 (4.871)	0.548	−3.362 (0.883)	<0.001	−4.204 (2.192)	0.055
AIC	1115.778	1114.035	1041.205			
Adjusted-BIC	1118.348	1116.604	1043.775			
CFI	0.981	0.985	1.000			

*Note*: *N* = 222 adolescents; number of neighborhoods = 45; sex: 0—male, 1—female; nativity: 0—United States, 1—Mexico.

Abbreviations: AIC, Akaike information criterion; BIC, Bayesian information criterion.

## Data Availability

The data that support the findings of this study are available on request from the corresponding author. The data are not publicly available due to privacy or ethical restrictions.

## Footnotes

1 The level 2 outcome (i.e., hair cortisol) in the multilevel analysis is the intercept of the level 1 hair cortisol, which is the hair cortisol after accounting for the level 1 individual difference within each neighborhood.
